# Effects of multisensory interventions initiated during NICU hospitalization on neurodevelopment in preterm infants: a systematic review and meta-analysis

**DOI:** 10.3389/fped.2026.1904270

**Published:** 2026-08-25

**Authors:** Rui Ren, Xinyi Men, Ming Xing Ma, Jun Guang Zhang

**Affiliations:** 1 Department of Pediatrics, Fengfeng General Hospital of North China Medical & Health Group, HanDan, China; 2 Department of Pediatrics, The Second Hospital of Hebei Medical University, Shijiazhuang, China

**Keywords:** meta-analysis, multisensory intervention, neurodevelopment, nursing care, preterm infant

## Abstract

**Background:**

This systematic review and meta-analysis aimed to evaluate the impact of multisensory interventions primarily initiated during NICU hospitalization or before discharge on neurodevelopment in preterm infants, informing clinical practice. While most interventions were delivered during the NICU stay, a minority involved post-discharge continuation through parental care plans. The study was conducted in strict accordance with the Preferred Reporting Items for Systematic Reviews and Meta-Analyses (PRISMA) guidelines and was registered with PROSPERO (CRD420251090728).

**Methods:**

Randomized controlled trials (RCTs) comparing the effects of multisensory intervention vs. standard neonatal care on neurological outcomes were included. The majority of interventions were delivered during NICU hospitalization; two studies involved post-discharge continuation through parental-implemented care plans. The intervention group received stimuli targeting two or more sensory modalities, while the control group received standard care or standard care supplemented with a single sensory intervention.

**Results:**

A total of 13 studies meeting the eligibility criteria were included in the meta-analysis. The pooled statistical analysis revealed that preterm infants receiving multisensory intervention exhibited significantly better neurodevelopmental outcomes compared to those receiving standard care or unisensory intervention (SMD = 0.96, 95% CI: 0.47 1.45, *P* < 0.00001). Subgroup analysis showed no statistically significant difference in intervention effects between assessments conducted at 3 months and beyond 3 months after intervention (Chi^2^ = 2.39, *I*^2^ = 58.2%, *P* = 0.12).

**Conclusions:**

The current evidence suggests that multisensory intervention initiated during the neonatal period (NICU hospitalization and early postnatal period) is an effective and safe approach for promoting neurodevelopment in preterm infants. However, due to the clinical heterogeneity observed among the included studies and the limited number of studies with post-discharge follow-up, further research is warranted to evaluate optimal timing, duration, and long-term effects across the continuum of care.

## 1 Introduction

The World Health Organization (WHO) defines preterm birth as any live birth that occurs before 37 weeks of gestation (1). Globally, approximately 14.9 million preterm births occur annually out of an estimated 135 million live births. Although the rate of births before 37 weeks varies significantly between countries, the associated morbidity and mortality represent a substantial burden (2). Global data from 2020 indicated that preterm birth complications were responsible for 73.4% of the 2.4 million neonatal deaths (3). Preterm birth is a leading cause of death in children under five years of age and a primary cause of neurodevelopmental disabilities worldwide, constituting a major public health challenge (4). Due to a significantly shortened gestation, preterm infants have underdeveloped physiological functions, making them highly susceptible to a range of serious complications. Compared to term infants, they face a significantly higher risk of neurodevelopmental impairments such as cerebral palsy and intellectual delay (5). Although advances in neonatal intensive care have markedly improved survival rates, issues related to morbidity and long-term quality of life persist. Therefore, early intervention is crucial for supporting optimal neurological development during this critical early life period and for laying the foundation for improved quality of life (6).

Since the 1960s, researchers have developed various interventions aimed at improving the growth and neurodevelopment of preterm infants, primarily involving environmental modifications, control of adverse stimuli (such as excessive light and noise), and the provision of positive sensory experiences (7). Based on the principles of neuronal plasticity and cortical reorganization, sensory stimulation, as an intervention delivered via peripheral input, can promote brain organization (8). Studies by Cristóbal Cañadas et al. (9) and Zengin et al. (10) on unisensory interventions, such as Kangaroo Mother Care (KMC), have demonstrated positive effects on physiological parameters, pain responses, and maternal milk production in preterm infants. However, descriptions of their impact on neurological outcomes remain limited. Taneja et al. (11) also noted that while KMC contributes to improved survival rates in preterm infants, it does not demonstrate significant benefits for neurodevelopment. Furthermore, many researchers have proposed simulating the intrauterine environment and utilizing complementary multisensory stimulation to maintain and promote the development of preterm infants (12). Nevertheless, the concurrent application of multisensory interventions has been relatively scarce.

Currently, multisensory intervention is increasingly becoming a focus of attention. The development of this intervention model aligns with the maturation of cortico-thalamic afferents to cortical multisensory areas (13). From a neurobiological perspective, Hebbian theory posits that coordinated, simultaneous activation across multiple sensory pathways strengthens synaptic connections through experience-dependent plasticity—a process that is particularly robust in the developing brain of preterm infants (14). From a developmental systems standpoint, the Synactive Theory (15) emphasizes that the autonomic, motor, and state subsystems of preterm infants are highly interdependent; appropriately modulated multisensory input can support cross-subsystem regulation, thereby facilitating behavioral organization while maintaining physiological stability. Furthermore, environmental enrichment theory suggests that complex and varied sensory experiences promote dendritic arborization and neurogenesis in the developing cortex. Integrating two or more sensory inputs, such as visual, auditory, tactile, and vestibular stimuli, provides systematic stimulation for preterm infants, thereby promoting neurodevelopment (16). Its core objective is to replicate intrauterine conditions early on to safeguard and accelerate the development of preterm infants while minimizing exposure to harmful environmental stimuli (7). Multiple studies support the positive significance of multisensory intervention in clinical practice. Research by Campbell-Yeo et al. (6) demonstrated that preterm infants receiving multisensory intervention in addition to standard care exhibited more favorable neurological outcomes at discharge. The recently proposed Sensory Development Care Map (SDCM) by Sampson et al. (17) offers clear, developmentally appropriate guidance by visually indicating when to protect or how to stimulate each sense. Furthermore, theories such as the Enriched Environment and the Synactive Model of Infant Development posit that multisensory stimulation has more significant neurological effects compared to unisensory stimulation (18). Collectively, this evidence underscores the critical role of multisensory intervention in improving neurological outcomes for preterm infants, highlighting its importance and value for broader clinical implementation (19, 20).

Based on the above, there is currently a lack of systematic reviews and quantitative analyses on the effects of multisensory interventions initiated during the neonatal period (NICU hospitalization and early postnatal period) on the neurodevelopment of preterm infants globally. This study employs systematic review and meta-analysis methods to comprehensively evaluate the efficacy of multisensory interventions delivered during hospitalization or with post-discharge continuation and standardize intervention protocols, aiming to provide an evidence-based foundation for developing precise neurodevelopmental intervention strategies for preterm infants during this critical early window.

## 2 Methods

This study has been registered on the PROSPERO platform (Registration number: CRD420251090728).

### 2.1 Search strategy

A systematic and comprehensive literature search was performed across four electronic databases: PubMed, Web of Science, Embase, and the Cochrane Library. The search scope covered all records from the inception of each database until June 2025. The objective of the search was to identify all randomized controlled trials (RCTs) that investigated the effects of multisensory intervention or multisensory stimulation on neurological outcomes in preterm infants. To minimize bias, two reviewers independently conducted the literature search and study selection process. The search strategy was designed using a combination of Medical Subject Headings (MeSH) terms and free-text terms, connected by Boolean operators (“AND” and “OR”). The core search concepts included “Multisensory,” “Intervention,” “Infant, Premature,” and “Neurologic Manifestations.” The specific search strategy was tailored to the requirements of each database. As an example, the detailed search strategy for PubMed was provided in Supplementary Material 1.

### 2.2 Eligibility criteria

#### 2.2.1 Definition of multi-sensory intervention

For this meta-analysis, a multimodal multisensory intervention was strictly defined as structured stimulation targeting two or more distinct sensory systems simultaneously or sequentially and provided alongside routine NICU care (13). The five primary sensory modalities were tactile, auditory, visual, vestibular, and gustatory-olfactory stimulation. Interventions were categorized as multisensory only when they integrated two or more sensory inputs, excluding single-sensory stimulation. The majority of eligible interventions began during NICU hospitalization; post-discharge continuation was permitted but not required. Only two included studies involved interventions initiated or continued after hospital discharge through parental care plans (21, 24). Therefore, this review primarily captures the effects of multisensory interventions delivered during the neonatal hospitalization period, with limited generalizability to exclusively home-based, long-term interventions. Qualified protocols included combined tactile massage, sensory-enriched kangaroo care, music/parental voice auditory stimulation, Auditory, Tactile, Visual, and Vestibular stimulus (ATVV) stimulation, the Supporting and Enhancing NICU Sensory Experiences (SENSE) program, the Multimodal Stimulation (MMS), and five-integrated sensory stimulation.

Although these interventions employed diverse protocols, they share three core conceptual commonalities justifying their inclusion in a single meta-analysis: (1) Structured stimulation beyond standard care: all provided organized, intentional sensory input rather than routine nursing care; (2) Multimodal integration: all targeted two or more distinct sensory systems (tactile, auditory, visual, vestibular, or gustatory-olfactory) either simultaneously or sequentially; and (3) Neurodevelopmental aim: all were designed to promote brain organization and neurodevelopmental outcomes through enriched environmental stimulation, consistent with theories of developmental neuroplasticity. Based on these shared characteristics, the included interventions were conceptually categorized into three approaches: (i) sensory-enriched kangaroo care (combining skin-to-skin contact with additional auditory, vestibular, or gustatory input); (ii) structured multisensory stimulation programs (systematic application of multiple sensory modalities through standardized protocols such as ATVV, SENSE, MMS, and five-integrated sensory stimulation); and (iii) post-discharge home-based multisensory interventions (parental training for continued stimulation after hospital discharge).

#### 2.2.2 Inclusion criteria

Studies were included if they met the following criteria: (1) Participants: Preterm infants with a gestational age of less than 37 weeks who are medically stable. (2) Intervention: The experimental group received a multisensory intervention (either alone or in combination with routine care). (3) Comparison: The control group received standard care in the Neonatal Intensive Care Unit (NICU). (4) Outcomes: The primary outcomes included measures of neurological development. (5) Study Design: Randomized controlled trials (RCTs).

#### 2.2.3 Exclusion criteria

Studies were excluded for the following reasons: (1) The full text did not provide clear results from multivariate analysis. (2) Duplicate publications. (3) Inability to access the full text or essential data. (4) Non-original research designs, including reviews, conference abstracts, case reports, and dissertations. (5) Studies that failed to report participant inclusion/exclusion criteria, lacked clearly defined outcome measures, or had obvious methodological flaws.

### 2.3 Study selection and data extraction

The literature selection and data extraction processes were conducted independently by two reviewers who had received formal training in systematic review methodology. The process consisted of two stages: Primary Screening: Titles and abstracts of all retrieved records were screened to exclude those that were clearly irrelevant to the review topic. Full-text Review: The full texts of the remaining articles were thoroughly assessed against the predefined eligibility criteria to determine final inclusion. Any disagreements between the two reviewers at either stage were resolved through discussion or, if necessary, by consulting a third reviewer for a final decision.

Data were extracted from the included studies using a standardized form based on the Cochrane Handbook for Systematic Reviews of Interventions (22). The extracted information included the first author, year of publication, country, sample size, details of the multisensory intervention and control groups, follow-up duration, and all relevant outcome measures. To enhance transparency regarding clinical heterogeneity, two reviewers independently characterized each intervention according to: (1) sensory modalities involved (tactile, auditory, visual, vestibular/kinesthetic, olfactory/gustatory); (2) intervention provider (parents, nurses, trained therapists, or research team); (3) frequency and duration of sessions; and (4) timing of delivery (during NICU hospitalization, post-discharge, or both). Interventions were classified as “multisensory” only when they integrated two or more sensory modalities beyond standard care. Discrepancies in classification were resolved through discussion until consensus was reached.

### 2.4 Risk of bias assessment

The methodological quality and risk of bias of the included studies were independently assessed by two reviewers. An appropriate critical appraisal tool was selected based on the study design. The reviewers used this tool to judge the risk of bias for each study, categorizing it as “low”, “high”, or “unclear”. The two reviewers initially performed the assessments independently, followed by a cross-checking process. Any discrepancies in their judgments were resolved through discussion until a consensus was reached. If necessary, a third reviewer was consulted to arbitrate unresolved disagreements.

### 2.5 Statistical analysis

Meta-analysis was performed using Review Manager (RevMan) software, version 5.4. Heterogeneity among the included studies was assessed using the Chi-square test (Q test) and the *I*^2^ statistic. A significance level of *P* < 0.05 for the Chi-square test and an *I*^2^ value greater than 75% were considered indicative of substantial heterogeneity (23). Based on the heterogeneity results, the choice of the analytical model was determined as follows: A fixed-effect model was employed when no significant heterogeneity was present (*P* ≥ 0.05 and *I*^2^ ≤ 50%). A random-effects model was used when significant heterogeneity was detected (*I*^2^ ≥ 50). For continuous outcomes, the mean difference (MD) along with the corresponding 95% confidence intervals (CIs) was calculated using the mean and standard deviation values extracted from the individual studies. Since the included studies utilized different assessment tools to measure neurological outcomes, separate meta-analyses were conducted for each specific instrument to ensure clinical and methodological homogeneity. The results of the meta-analyses are presented graphically using forest plots.

## 3 Results

### 3.1 Search strategy

**Figure 1:**
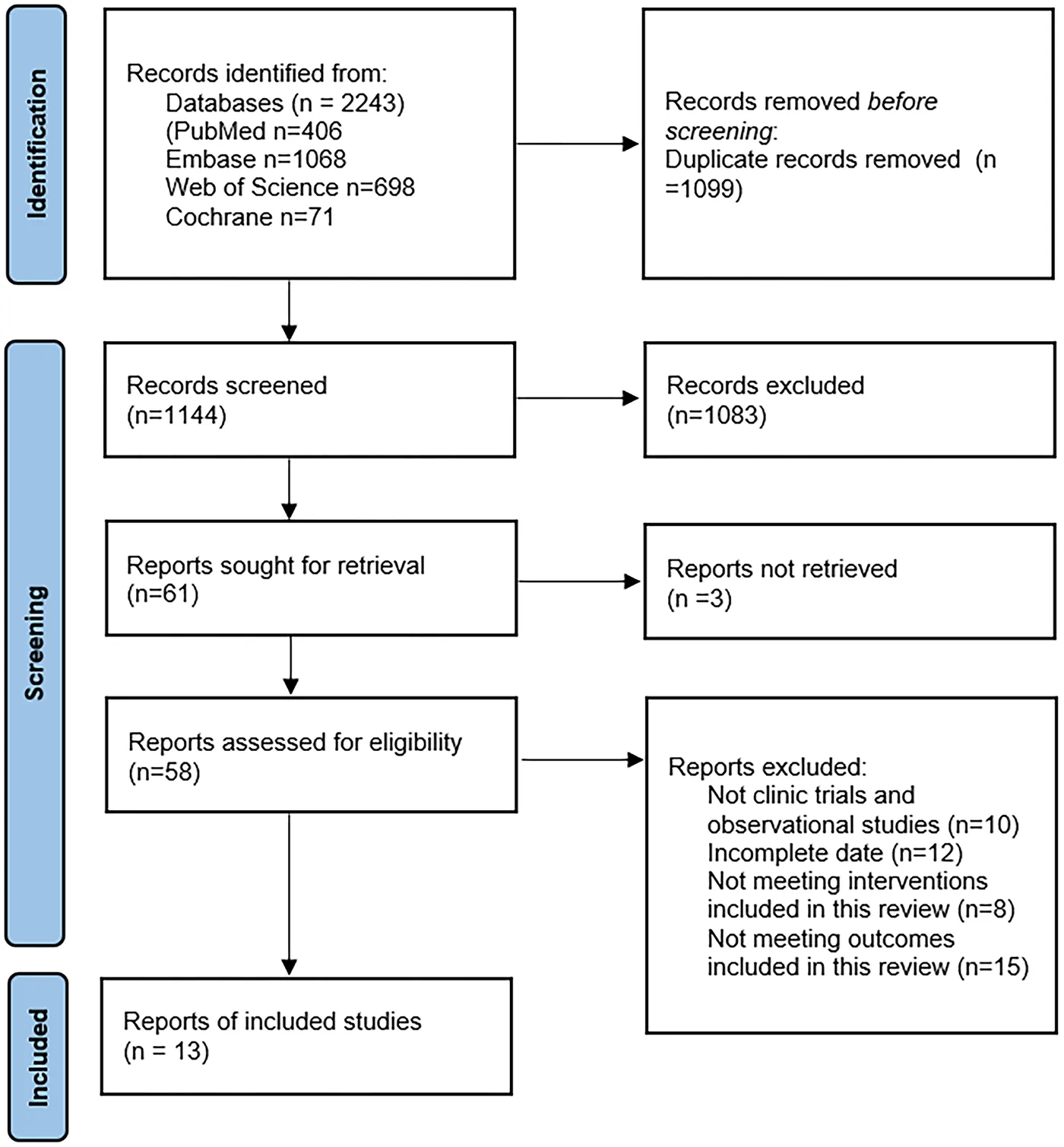
PRISMA flow diagram.

The initial systematic literature search identified a total of 2,243 records. After the removal of duplicates, 1,144 unique citations remained. Following a preliminary screening of titles and abstracts, 1,086 records were excluded due to irrelevance to the research topic or because they were review articles, conference abstracts, guidelines, or utilized ineligible study designs. This resulted in 58 articles being retrieved for a full-text review. After a detailed assessment of the full texts against the predefined eligibility criteria, 45 articles were excluded, culminating in the final inclusion of 13 English-language studies in the systematic review (7, 24–34). The complete study selection process is detailed in the PRISMA flow diagram (Figure 1).

### 3.2 Characteristics of included studies

A summary of the basic characteristics of the included studies is presented in Supplementary Material 2. The systematic review included 13 randomized controlled trials (RCTs) published from the inception of the databases until June 2025. The included studies were conducted across diverse geographical regions: three in China (21, 24, 34), three in Switzerland (25, 29, 30), two in the United States (27, 32), and one in Brazil (26), one in Egypt (31), New Zealand (28), India (33), and Iran (7). Regarding participant characteristics, seven studies enrolled preterm infants with a gestational age of ≤32 weeks (21, 25–27, 29–30, 32), while the remaining six studies included infants with a gestational age ranging from 28 to 36 weeks (7, 24, 28, 31, 33, 35). The interventions consisted of multisensory stimulation, defined as the application of two or more sensory modalities, provided in addition to standard neonatal care. Three studies implemented creative live music therapy during skin-to-skin contact (25, 29, 30), one study involved breastfeeding during kangaroo mother care (34), and the remaining nine studies employed integrated multisensory stimulation interventions (7, 21, 24, 26–28, 31–33). The interventions were administered by parents, nurses, or trained research team members. Ten studies delivered the intervention during the infant's hospital stay (7, 25–29, 31–34), while two studies involved parental care plans implemented after hospital discharge (21, 24).

### 3.3 Outcome measures and follow-up

Neurological outcomes were assessed using a variety of standardized tools. The Infant Neurological International Battery (INFANIB) was used in two studies (28, 33), and the Bayley Scales of Infant and Toddler Development, Third Edition (Bayley-III) was applied in two studies (29, 30). The other studies utilized different assessment tools, as detailed in Table 1. The follow-up duration for neurological assessment varied considerably. The shortest follow-up was at 6 days of corrected gestational age, assessed using the INFANIB by Aranha et al. (33), while the longest follow-up extended to 5 years, measured using the Kaufmann Assessment Battery, second edition (KABC-II) by Haslbeck et al. (30).

**Table 1:** Subgroup analysis.

Subgroup variable	Number of studies	Effect size (SMD, 95% Cl)	*I* ^2^	*P*-value for subgroup difference
Follow-up period				0.12
≤3 months	377	1.29 (0.55–2.03)	90%	0.0006
＞3 months	474	0.58 (0.06–1.10)	85%	0.03

### 3.4 Risk of bias of included studies

**Figure 2:**
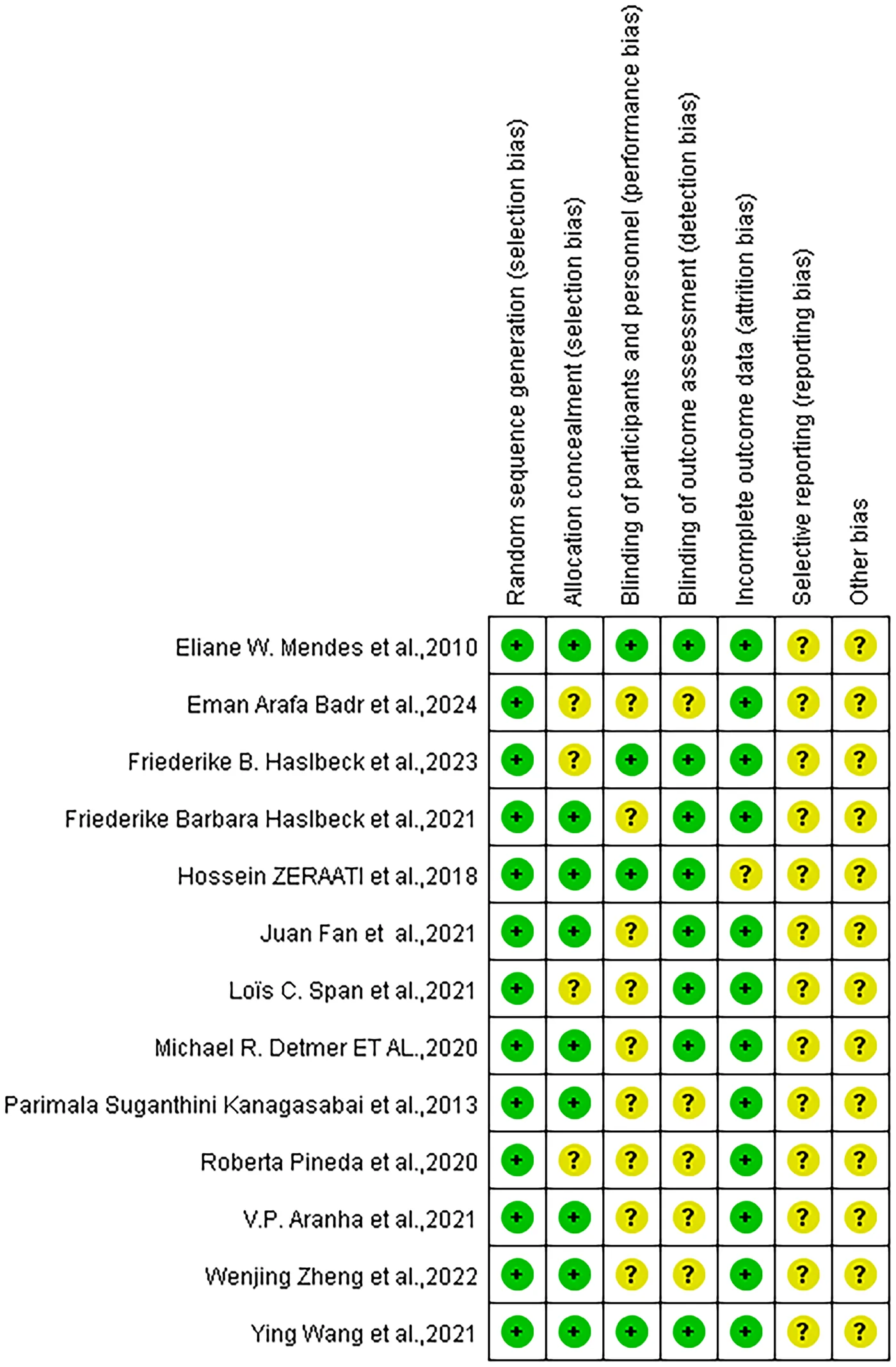
Summary of the risks of bias of the included studies.

**Figure 3:**
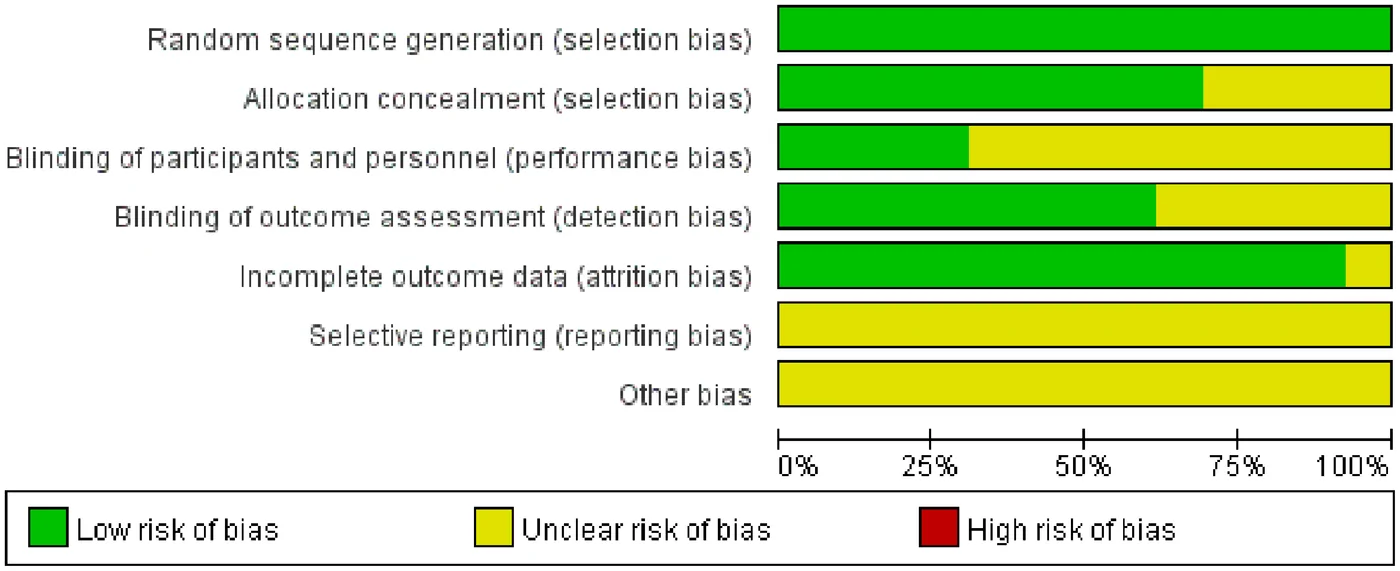
Risks of bias of the included studies.

The overall results of the risk of bias assessment for the included studies are summarized in Figure 2 (risk of bias summary) and Figure 3 (risk of bias graph). All included studies (*n* = 13) employed a randomized controlled trial design (7, 21, 24, 25–34). Regarding allocation concealment, nine studies (7, 21, 24, 26, 28, 29, 32–34) provided adequate descriptions and were judged as having a low risk of bias. Due to the nature of the interventions, blinding of participants and personnel was challenging. Only four studies (7, 26, 30, 34) were deemed to have a low risk of performance bias, primarily because of their specific study designs; for the remaining studies, the risk of performance bias was judged as unclear due to a lack of explicit information. In terms of blinding of outcome assessment (detection bias), eight studies (7, 21, 25, 26, 29–30, 32, 34) clearly stated that outcome assessors were blinded, resulting in a low risk of detection bias. All studies were assessed as having a low risk of bias for incomplete outcome data, as they provided complete reporting of their primary outcomes. For selective reporting and other potential sources of bias, the risk for most studies remained unclear due to insufficient information provided in the original publications. Potential publication bias was explored visually using funnel plots.

### 3.5 Meta-Analyses

**Figure 4:**
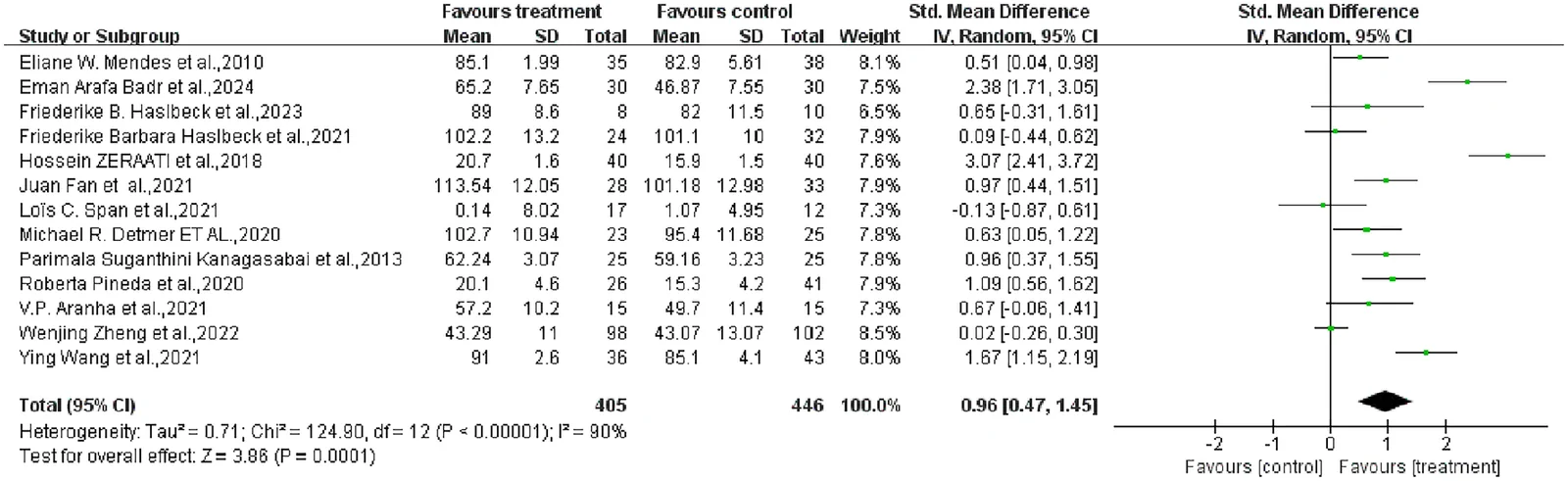
Forest plot showing the effects of multi-sensory intervention on the nervous system of premature infants.

Thirteen studies, involving a total of 851 preterm infants, reported on the effects of multisensory intervention on comprehensive neurological scores. A random-effects model meta-analysis was employed due to significant heterogeneity. The pooled results indicated that multisensory interventions had a statistically significant, positive effect on neurological outcomes compared to control interventions [Standardized Mean Difference (SMD) = 0.96, 95% CI: 0.47–1.45, *P* < 0.001]. However, considerable heterogeneity was observed among the studies (*I*^2^ = 90%, *P* < 0.001), suggesting substantial variation in the effect sizes. Consequently, these findings should be interpreted with caution. The forest plot for this analysis is presented in Figure 4.

### 3.6 Sensitivity analysis

A sensitivity analysis was performed to assess the robustness of the pooled results and to identify potential sources of the substantial heterogeneity. The analysis involved iteratively removing each included study and recalculating the overall effect size. The results indicated that the exclusion of any single study did not significantly alter the overall effect size or substantially reduce the heterogeneity, suggesting that no single study was the dominant source of the observed statistical heterogeneity.

### 3.7 Subgroup analysis

A subgroup analysis was performed based on the timing of follow-up assessment (≤3 months vs. >3 months post-intervention) to explore its potential influence on the outcomes. The results are detailed in Table 1.

#### 3.7.1 Subgroup of follow-up at ≤3 months

Seven studies were included in this subgroup, involving 181 participants in the intervention groups and 196 in the control groups. Due to high heterogeneity (*I*^2^ = 90%), a random-effects model was applied. The analysis revealed a significant positive effect of multisensory intervention on neurological outcomes, with a large effect size (Standardized Mean Difference, SMD = 1.29, 95% CI: 0.55–2.03; *Z* = 3.44, *P* = 0.0006).

#### 3.7.2 Subgroup of Follow-up at > 3 Months

Six studies were included in this subgroup, comprising 224 participants in the intervention groups and 250 in the control groups. A random-effects model was also used due to substantial heterogeneity (*I*^2^ = 85%). The analysis indicated a statistically significant, though moderate, positive effect (SMD = 0.58, 95% CI: 0.06–1.10; *Z* = 2.18, *P* = 0.03).

#### 3.7.3 Test for subgroup differences

A Chi-square test was conducted to compare the effect sizes between the two subgroups. The result indicated no statistically significant difference between the subgroups (Chi^2^ = 2.39, df = 1, *P* = 0.12; *I*^2^ = 58.2%).

The subgroup analysis suggests that multisensory intervention has a statistically significant positive effect on neurological outcomes in preterm infants, regardless of whether the assessment is conducted within 3 months or after 3 months post-intervention. The point estimate of the effect size appears larger in the earlier follow-up subgroup, but the difference between the two time points did not reach statistical significance. The substantial heterogeneity observed within each subgroup warrants cautious interpretation.

### 3.8 Publication bias

**Figure 5:**
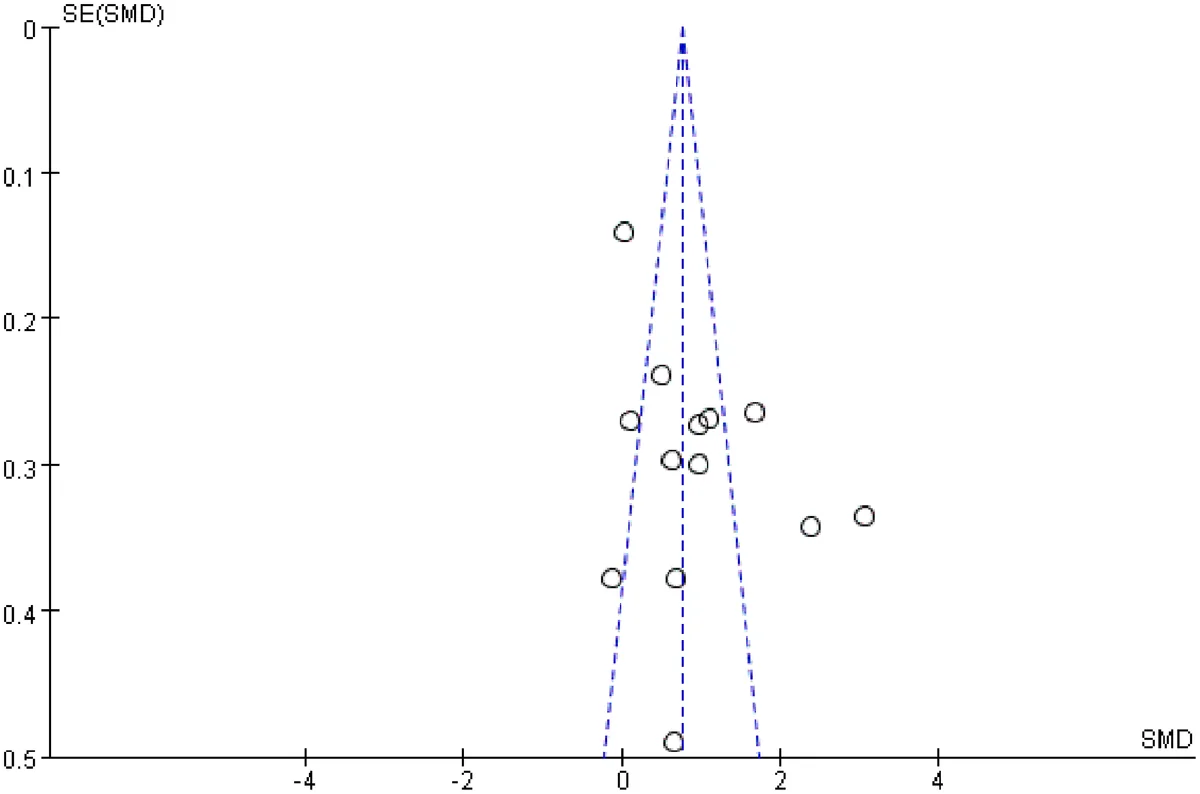
Funnel plot for publication bias of the effect of multisensory intervention on neurological outcomes in preterm infants.

Publication bias was assessed visually using a funnel plot (Figure 5). The distribution of the study points in the funnel plot showed asymmetry, deviating from the expected symmetrical inverted-funnel shape. In the absence of publication bias, studies are expected to be distributed evenly within the funnel. The observed asymmetry, with several study points located away from the center of the funnel, suggests the potential presence of publication bias. This may indicate that studies with non-significant or negative results might have been less likely to be published and were therefore missing from the current analysis, which could introduce bias into the pooled effect estimate.

## 4 Discussion

This meta-analysis investigated the effects of multisensory intervention on neurological outcomes in preterm infants during their NICU stay and after discharge. A total of 13 randomized controlled trials, involving 851 preterm infants, were included. The results demonstrate that, compared to standard NICU care, multisensory intervention is a safe and effective nursing approach that significantly improves neurological outcomes (overall *P* < 0.05). Subgroup analysis based on the timing of follow-up (≤3 months vs. >3 months) revealed no statistically significant difference in effect sizes between the two periods, suggesting that the positive influence of the intervention may be sustained over time. The high statistical heterogeneity observed may be attributable to variations in the implemented intervention across the included studies. Despite this heterogeneity, the present analysis strengthens the empirical evidence supporting the beneficial role of multisensory intervention on neurological development in preterm infants. A previous systematic review (35) examined the effectiveness of various interventions on early neurodevelopment, which included two studies on multisensory interventions (28). Our review builds upon this foundation by incorporating an additional 11 recent studies focused specifically on multisensory interventions and employing a comprehensive assessment using various neurobehavioral and developmental scales. This expansion enhances the comprehensiveness and reliability of the conclusions regarding the efficacy of multisensory interventions.

Multisensory intervention is an increasingly adopted neuroprotective measure, typically initiated during NICU stay. Its core objective is to simulate the intrauterine environment to reduce stress responses in preterm infants, aligning with the principles of “developmentally supportive care.” The theoretical foundation for multisensory intervention rests upon multiple complementary developmental frameworks. Als' Synactive Theory of Infant Development (36) posits that preterm infants actively organize their behavior through reciprocal interaction with environmental stimuli, emphasizing the modification of NICU environment and care practices to minimize stressors and provide protective support for brain and neurological development. Sensory Integration Theory (15) proposes that the central nervous system processes and integrates multisensory input to produce adaptive responses, with early sensory experiences fundamentally shaping the architecture of developing neural circuits. Dynamic Systems Theory (37) conceptualizes neurodevelopment as emerging from continuous interactions between the infant and nested environmental systems, highlighting how varied sensory experiences drive developmental transitions and self-organization. Ecological Systems Theory (38) underscores the NICU environment and family context as influential microsystems, supporting the integration of parental involvement in multisensory interventions. Finally, contemporary concepts of developmental neuroplasticity emphasize that early life represents a critical window during which sensory experiences induce lasting structural and functional brain changes through activity-dependent synaptogenesis, pruning, and myelination (39, 40). Collectively, these frameworks provide a robust, multidimensional conceptual basis for multisensory intervention. Evidence suggests that multisensory stimulation yields superior neurodevelopmental effects compared to unisensory interventions (18, 41, 42). The findings of this study corroborate the practical applicability of these theoretical frameworks, demonstrating that multisensory intervention is not only empirically feasible but also consistent with contemporary developmental care paradigms. The present study confirms that multisensory intervention effectively improves neurological outcomes in preterm infants, a finding consistent with previous research (43). This supports the view that, compared to unisensory stimulation or standard care, multisensory intervention offers distinct advantages for the physical and neurodevelopmental progress of preterm infants. Its efficacy is fundamentally rooted in its profound impact on key processes of early brain development. At the neurobiological level, the effectiveness of this intervention relies on the high degree of neuronal plasticity characteristic of early life (44). The integrated input of multiple sensory stimuli not only promotes widespread synaptogenesis and pruning via thalamic convergence but also directly drives axonal myelination and the structural optimization of neural networks (45, 46). These processes form the foundation for functional brain maturation. This mechanism is critical for enhancing information processing efficiency (1), and its clinical significance is directly manifested in promoting compensatory maturation of brain structure in preterm infants. This may effectively mitigate long-term risks associated with preterm birth, particularly in higher-order brain functions such as learning, memory, and emotional regulation (16). The clinical benefits of multisensory intervention also extend to the regulation of overall physiological rhythms and parent-infant interaction. Facilitating the integration of multimodal sensory information, it supports the more mature development of sleep-wake cycle regulatory centers in the brain (47), thereby creating a more favorable internal environment for somatic growth and brain development. Furthermore, multisensory intervention establishes a synchronous, non-verbal dialogue between the caregiver and the preterm infant (48). This resonance of emotional and behavioral states is clinically crucial for fostering secure parent-infant attachment and promoting the development of self-awareness and social competencies in the infant. Therefore, multisensory intervention is not merely a neurodevelopmental support strategy but a comprehensive clinical rehabilitation approach encompassing brain function maturation, physiological rhythm regulation, and socio-emotional development. It holds significant potential for improving the long-term prognosis of preterm infants.

Future research should focus on developing systematic and standardized long-term multisensory intervention protocols, with parents positioned as central active participants to fully leverage their crucial role in early parent-infant interactions. Current evidence lacks exploration of adaptive intervention pathways tailored to preterm infants of different corrected ages and risk stratification. Additionally, high-quality implementation science research is urgently needed to systematically analyze the factors influencing the integration of multisensory interventions into routine clinical care. This includes examining aspects such as healthcare professional training, family engagement, and the allocation of necessary medical resources. By scientifically evaluating the scalability and implementation outcomes of these interventions, we can effectively bridge the gap between research evidence and clinical practice, ultimately improving the long-term developmental outcomes of preterm infants.

## 5 Limitations

This analysis has several limitations that should be considered when interpreting the findings. Firstly, the number of included studies was relatively small (*n* = 13), and the overall sample sizes were limited, which constrains the precision and generalizability of the effect size estimates for some outcomes. The main limitations include: (1) significant heterogeneity in the specific components and protocols of the multisensory interventions across the 13 studies, which involved eight distinct combinations of sensory stimuli; (2) measurement heterogeneity resulting from the use of diverse assessment tools to evaluate neurological outcomes; (3) a notable lack of reported safety data, such as potential adverse effects like abnormal heart rate patterns or increased irritability during or following the interventions; and (4) the scope of this review is primarily limited to interventions initiated during NICU hospitalization or before discharge, with only two studies involving post-discharge implementation. Consequently, our findings may not be generalizable to long-term, exclusively home-based multisensory intervention programs or to the full continuum of care from hospital to home.

## 6 Conclusion

Based on the current body of evidence, this analysis indicates that multisensory intervention exerts a positive influence on neurobehavioral development, with significant effects observed across varying intervention durations and follow-up periods. The consistent integration of multisensory interventions into clinical practice, spanning both the NICU hospitalization and subsequent home-based follow-up care, remains a significant and persistent challenge.
