# Supplementary material for: Effects of multisensory interventions initiated during NICU hospitalization on neurodevelopment in preterm infants: a systematic review and meta-analysis
Source: Front Pediatr. 2026 Aug 25;14:1904270. doi: 10.3389/fped.2026.1904270 (PMC13550988; doi:10.3389/fped.2026.1904270)
Supplement: Supplementary file 1 [file Table1.docx]

1. PubMed

("Multisensory"[Title/Abstract] OR "Multi-sensory"[Title/Abstract] OR "Multimodal sensory"[Title/Abstract] OR "Sensory stimulation"[Title/Abstract] OR "Sensory input"[Title/Abstract] OR "Touch"[Title/Abstract] OR "Massage"[Title/Abstract] OR "Tactile stimulation"[Title/Abstract] OR "Kangaroo care"[Title/Abstract] OR "Skin-to-skin contact"[Title/Abstract] OR "SSC"[Title/Abstract] OR "Non-nutritive sucking"[Title/Abstract] OR "Pacifier"[Title/Abstract] OR "Music therapy"[Title/Abstract] OR "Auditory stimulation"[Title/Abstract] OR "Voice therapy"[Title/Abstract] OR "Visual stimulation"[Title/Abstract] OR "Eye contact"[Title/Abstract] OR "Vestibular stimulation"[Title/Abstract] OR "Rocking"[Title/Abstract]) AND ("Intervention"[Title/Abstract] OR "therap*"[Title/Abstract] OR "treat*"[Title/Abstract] OR "Program"[Title/Abstract] OR "Stimulation"[Title/Abstract] OR "train*"[Title/Abstract] OR "manage*"[Title/Abstract] OR "Approach"[Title/Abstract]) AND ("infant, premature"[MeSH Terms] OR "infant, low birth weight"[MeSH Terms] OR "infant, very low birth weight"[MeSH Terms] OR "premature infant*"[Title/Abstract] OR "preterm infant*"[Title/Abstract] OR "preterm newborn*"[Title/Abstract] OR "premature newborn*"[Title/Abstract] OR "low birth weight infant*"[Title/Abstract] OR "lbw infant*"[Title/Abstract] OR "vlbw infant*"[Title/Abstract] OR "elbw infant*"[Title/Abstract]) AND ("Neurologic manifestations"[MeSH Terms] OR "Neurodevelopmental disorders"[MeSH Terms] OR "Nervous system"[MeSH Terms] OR "neurolog*"[Title/Abstract] OR "neurodevelopment*"[Title/Abstract] OR "neural development*"[Title/Abstract] OR "brain development*"[Title/Abstract])

N=406

2. WOS

(TS=("Multisensory" OR "Multi-sensory" OR "Multimodal sensory" OR "Sensory stimulation" OR "Sensory input" OR "Touch" OR "Massage" OR "Tactile stimulation" OR "Kangaroo care" OR "Skin-to-skin contact" OR "SSC" OR "Non-nutritive sucking" OR "Pacifier" OR "Music therapy" OR "Auditory stimulation" OR "Voice therapy" OR "Visual stimulation" OR "Eye contact" OR "Vestibular stimulation" OR "Rocking") OR TI=("Multisensory" OR "Multi-sensory" OR "Multimodal sensory" OR "Sensory stimulation" OR "Sensory input" OR "Touch" OR "Massage" OR "Tactile stimulation" OR "Kangaroo care" OR "Skin-to-skin contact" OR "SSC" OR "Non-nutritive sucking" OR "Pacifier" OR "Music therapy" OR "Auditory stimulation" OR "Voice therapy" OR "Visual stimulation" OR "Eye contact" OR "Vestibular stimulation" OR "Rocking")) AND (TS=("Intervention" OR "Therap*" OR "Treat*" OR "Program" OR "Stimulation" OR "Train*" OR "Manage*" OR "Approach") OR TI=("Intervention" OR "Therap*" OR "Treat*" OR "Program" OR "Stimulation" OR "Train*" OR "Manage*" OR "Approach")) AND (TS=("Premature infant*" OR "Preterm infant*" OR "Preterm newborn*" OR "Premature newborn*" OR "Premature neonate*" OR "Preterm neonate*" OR "Low birth weight infant*" OR "LBW infant*" OR "VLBW infant*" OR "ELBW infant*") OR TI=("Premature infant*" OR "Preterm infant*" OR "Preterm newborn*" OR "Premature newborn*" OR "Premature neonate*" OR "Preterm neonate*" OR "Low birth weight infant*" OR "LBW infant*" OR "VLBW infant*" OR "ELBW infant*")) AND (TS=("Neurolog*" OR "Neurodevelopment*" OR "Neural development*" OR "Brain development*" OR "Nervous system") OR TI=("Neurolog*" OR "Neurodevelopment*" OR "Neural development*" OR "Brain development*" OR "Nervous system"))

N=698

3. EMBASE

('multisensory' OR 'multi-sensory' OR 'sensory stimulation'/exp OR 'touch'/exp OR 'massage'/exp OR 'kangaroo care'/exp OR 'skin to skin contact'/exp OR 'nonnutritive sucking'/exp OR 'pacifier'/exp OR 'music therapy'/exp OR 'auditory stimulation'/exp OR 'visual stimulation'/exp OR 'vestibular stimulation'/exp OR multisensory:ti,ab,kw OR 'multi sensory':ti,ab,kw OR 'sensory input':ti,ab,kw OR touch:ti,ab,kw OR massage:ti,ab,kw OR 'tactile stimulation':ti,ab,kw OR 'kangaroo care':ti,ab,kw OR 'skin to skin contact':ti,ab,kw OR ssc:ti,ab,kw OR 'non nutritive sucking':ti,ab,kw OR pacifier:ti,ab,kw OR 'music therapy':ti,ab,kw OR 'auditory stimulation':ti,ab,kw OR 'voice therapy':ti,ab,kw OR 'visual stimulation':ti,ab,kw OR 'eye contact':ti,ab,kw OR 'vestibular stimulation':ti,ab,kw OR rocking:ti,ab,kw) AND ('intervention'/de OR 'therapy'/exp OR 'training'/de OR 'management'/de OR intervention:ti,ab,kw OR therapy:ti,ab,kw OR treatment:ti,ab,kw OR program*:ti,ab,kw OR stimulation:ti,ab,kw OR training:ti,ab,kw OR management:ti,ab,kw OR approach:ti,ab,kw) AND ('prematurity'/exp OR 'low birth weight'/exp OR 'premature infant':ti,ab,kw OR 'preterm infant':ti,ab,kw OR 'preterm newborn':ti,ab,kw OR 'premature newborn':ti,ab,kw OR 'preterm neonate':ti,ab,kw OR 'premature neonate':ti,ab,kw OR 'low birth weight infant':ti,ab,kw OR 'lbw infant':ti,ab,kw OR 'vlbw infant':ti,ab,kw OR 'elbw infant':ti,ab,kw) AND ('nervous system'/exp OR 'neurologic disease'/exp OR 'neurodevelopment'/de OR neurolog*:ti,ab,kw OR neurodevelopment*:ti,ab,kw OR 'neural development':ti,ab,kw OR 'brain development':ti,ab,kw OR 'nervous system':ti,ab,kw)

N=1068

4. Cochrane

ID	Search	Hits

#1	(Multisensory OR "multi sensory" OR "sensory stimulation" OR "sensory input" OR Touch OR Massage OR "tactile stimulation" OR "kangaroo care" OR "skin to skin contact" OR SSC OR "non nutritive sucking" OR Pacifier OR "music therapy" OR "auditory stimulation" OR "voice therapy" OR "visual stimulation" OR "eye contact" OR "vestibular stimulation" OR Rocking): ti, ab,kw	24668

#2	(Intervention OR Therapy OR Treatment OR Program OR Stimulation OR Training OR Management OR Approach): ti, ab,kw	1704556

#3	("Premature infant" OR "Preterm infant" OR "Preterm newborn" OR "Premature newborn" OR "Preterm neonate" OR "Premature neonate" OR "Low birth weight infant" OR "LBW infant" OR "VLBW infant" OR "ELBW infant"): ti, ab, kw	2465

#4	Neurologic* OR Neurodevelopment* OR "neural development" OR "brain development" OR "nervous system"	75099

#5	#1 AND #2 AND #3 AND #4	71

N=71
