# Supplementary material for: Effects of multisensory interventions initiated during NICU hospitalization on neurodevelopment in preterm infants: a systematic review and meta-analysis
Source: Front Pediatr. 2026 Aug 25;14:1904270. doi: 10.3389/fped.2026.1904270 (PMC13550988; doi:10.3389/fped.2026.1904270)
Supplement: Supplementary file 2 [file Table2.docx]

Supplement 2. Characteristics of included studies in the systematic review

| **Author, Year** | **Country** | | **N (Exp/Con)** | | **Tactile** | | **Auditory** | **Visual** | **Vestibular/Kinesthetic** | **Olfactory/Gustatory** | **Provider** | | **Frequency/Duration** | **Intervention time point** | | **Outcome** |
| --- | --- | --- | --- | --- | --- | --- | --- | --- | --- | --- | --- | --- | --- | --- | --- | --- |
| Fan et al., 2021 | | China | 73/33 | Whole-body massage;  kangaroo care | | Hearing-vision integrated training | | Eye contact; visual tracking | Kangaroo care position | — | Parents | Daily; 120 days | | Post-discharge | TIMP | |
| Badr et al., 2024 | | Egypt | 30/30 | warm palms in a sequence of chest, upper limbs, and lower limbs in a supine position, and  then returning (3min) | | mother’s  heartbeats (3min) | | Black and white visual stimulation  (3min) | Placed supine in a hammock, gradually elevated to semi-sitting, then lowered back to supine. (3min) and Supine position; passive flexion/extension of upper and lower limbs(3min) | Olfactory  Stimulation  (12min) | Nurses | 30 min/day; 2 weeks | | During NICU | NNES | |
| Haslbeck et al., 2023 | | Switzerland | 12/19 | Skin-to-skin contact | | Creative music therapy | | — | Holding/positioning | — | Parents/Therapists | 20 min, 2-3×/week until discharge | | During NICU | KABC-II | |
| Mendes et al., 2010 | | Brazil | 35/38 | Temporal, frontal, periorbital, nasal, and perilabial regions (face); lateral sides of upper/lower limbs. | | — | | — | Passive flexion/extension of upper/lower limbs, one limb at a time, up to 3 reps per joint (wrist, elbow, ankle, knee); one hand supports limb, other performs movements | — | Mother | 4 times/day, 15 min each2 years | | During NICU | BSID-II | |
| Pineda et al., 2020 | | USA | 30/50 | skin-to-skin, touch, holding | | human speech, music | | dim or cycled light | movement, rocking/transfers | scent cloth, close maternal contact | Parents/Nurses | 2×/day, 15min/session; until discharge | | During NICU | HNNE | |
| Kanagasabai et al., 2013 | | New Zealand | 25/25 | Gentle stroking massage for 3 min in sequence: chest → upper limbs → lower limbs (supine).(3min) | | Soft lullaby(3min) | | Black and white visual stimulation card(3min) | Gentle horizontal and  vertical rocking(3min) | — | Nurses | 12min/session, 5 days/week; at term equivalent age | | During NICU | INFANIB | |
| Detmer et al., 2020 | | USA | 38/38 | Stroking body areas in cephalocaudal & proximodistal order: head → back → neck → arms → chest → abdomen → legs → cheeks → forehead → nose-to-ear (over blanket if swaddled). | | Gentle quiet live lullaby singing (males); gentle quiet live lullaby singing with arpeggiated guitar accompaniment (females).(15–30 min/sessin, 1-2×/week) | | Eye contact from the music therapist | Front-to-back rocking in rocking chair, synchronized with music tempo. | — | Therapists | 20 min/session,2-3×/week; untildischarge | | During NICU | MSEL | |
| Aranha et al., 2021 | | India | 15/15 | Gentle stroking massage (3min) | | Maternal voice  (3min)  stimulation | | Black and white card(3min) | Guided range of motion (upper/lower limbs); antigravity movements in prone (neck/spinal extension); antigravity movements in supported sitting; upright positioning. (12min) | Stroking cheeks, lips, jaw, tongue; rubbing gums (3min) | Medical team | 5 sessions, 30 min/session | | During NICU | INFANIB | |
| Span et al., 2021 | | Switzerland | 12/17 | Kangaroo care (30min) | | Live-performed music therapy (6 sessions, 15 min/session) | | — | Kangaroo care positioning | — | Parents/Therapists | 7 days after birth | | During NICU | GMA | |
| Haslbeck et al., 2021 | | Switzerland | 23/29 | Skin-to-skin contact | | Creative music therapy | | — | Holding/positioning | — | Parents/Therapists | 20 min/session, 2–3×/week, until discharge | | During NICU | Bayley-III | |
| Zheng et al., 2022 | | China | 98/102 | Gentle touch | | Maternal Voice | | Eye contact | — | Breastmilk/formula/food odor of caregiver) | Parents | ≥10 min/session, ≥3×/day; 6 months | | Post-discharge | ASQ-3 | |
| Wang et al., 2021 | | China | 36/43 | Kangaroo mother care | | — | | — | Kangaroo care positioning | Direct breastfeeding | Mothers | 2.5 h/day, until discharge; 6 months | | During NICU | NBNA | |
| Zeraati et al., 2018 | | Iran | 40/40 | Sequential massage: chest → upper limbs → lower limbs, in supine position(3min) | | Soft lullaby (3min) | | Black and white visual stimulation card (3min) | Gentle horizontal and vertical rocking (3min) | — | Nurses | Start at 48h postnatal,12 min/session, 5×/week; until discharge | | During NICU | NBS | |

Note: Test of Infant Motor Performance (TIMP), The Neonatal Neurobehavioral Examination Scale (NNES), Kaufmann Assessment Battery, second edition (KABC-II), Bayley Scales of Infant Development, Second Edition (BSID-II), Hammersmith Neonatal Neurological Evaluation (HNNE), Infant Neurological International Battery (INFANIB), Mullen Scales of Early Learning: AGS Edition 36 (MSEL), Infant Neurological International Battery (INFA NIB), General Movement Assessment (GMA), Bayley Scales of Infant and Toddler Development, Third Edition (Bayley-III), Ages & Stages Questionnaires®, Third Edition(ASQ-3), Neonatal Behavioral Neurological Assessment (NBNA), New Ballard Score(NBS)
